# Protective effect of Erythropoietin on renal injury induced in rats by four weeks of exhaustive exercise

**DOI:** 10.1186/1471-2369-14-130

**Published:** 2013-06-24

**Authors:** Xixiu Lin, Chonghe Jiang, Ziqiang Luo, Shulin Qu

**Affiliations:** 1 Department of Physiology, Central South University, Changsha 410013, China; 2 Kidney Center, Qingyuan City People’s Hospital of Jinan University, Guangdong 511 518, China; 3 Medical College of Hunan Normal University, Changsha 410012, China

**Keywords:** Chronic Injury, Erythropoietin, Exhaustive Exercise, Kidney, Rat

## Abstract

**Background:**

The protective effect of Erythropoietin (EPO) analogue rHuEPO on acute renal injury induced by exhaustive exercise had been reported. The purpose of this study is to probe into the protective effect of EPO on chronic renal injury induced by repeated exhaustive exercise for four weeks.

**Methods:**

Eighty adult male Sprague–Dawley rats were used in this experiment. The animals were randomly allocated to one of four groups: control (C), exhaustive exercise test (ET), ET plus EPO pre-treatement (ET+EPO) and ET+EPO plus LY294002 pretreatment (ET+EPO+LY).

**Results:**

Compared with the rats in control group, there was considerable damage in kidney cells in rats of ET group as revealed by histological and ultrastructural examinations. However, treatment with EPO during the training, the exhaustive running distance was significant increased (*P* < 0.01), and the pathological changes of kidney cell were much less compared with those of rats without EPO intervention. When LY294002, a specific inhibitor of phospholipids phthalocyanine inositol 3-kinase, was added to the EPO treated rats, the injury changes of renal cell were becoming more pronounced.

**Conclusions:**

The protective effect of EPO on chronic renal injury induced by repeated exhaustive exercise was demonstrated in the present study. We proposed that the effect could be due to inhibiting the cell apoptosis and blocking the formation of interstitial fibrosis via activation of the PI3K/Akt pathway, thus plays role in the endogenous protection of the kidney injury.

## Background

Erythropoietin (EPO) belongs to members of multi-functional cytokine super-family and provides protection for various organs [1-3]. In the brain and spinal cord of the rats, EPO is increased in the condition of oxygen deprivation and mediated by hypoxia inducible factors [4]. By blocking the transduction of apoptosis signal, EPO facilitates the production of apoptosis protein inhibitor and exerts neuro-protective effects. EPO was also shown to have inhibition effect of apoptosis in the retina of the rats [5-7]. It has been proved that EPO is a kind of hemopoietic growth factor with independent efficiency in hemopoiesis [8]. For example, with the functions of anti-inflammatory, antioxidation, antiapoptosis, EPO exerts the protective effects on central nervous system, kidney, retina etc. [9], and prevents the harms caused by acute ischemia, chronic ischemia, or hypoxia. So far, the EPO protective effect and the work mechanism of chronic renal injury resulted from repeated exhaustive exercise have rarely been reported.

High-intensity work, military training and competitive sports will inevitably cause overload or hypoxia. Long-term high-intensity exercise or overload that exceeds the endurance of the body will cause qualitative change from physiologic stimuli to pathologic stimuli. Eventually, the systemic function will be affected, such as rhabdomyolysis and injuries of heart, liver and neural system. Kidney is one of the vulnerable organs suffered from the high-intensity exercise or overload training, manifesting as cystorrhexis, hematuria, proteinuria, acute renal failure etc. Therefore, to prevent and treat the kidney and other organs injury resulted from overtraining are crucial tasks in sports medicine, modern military medicine and clinical medicine. The present study was designed to determine the histological and ultrastructural changes in kidney cell of the rats after relatively long exhaustive exercise, evaluate the preventive and therapeutic effects of EPO on such a pathological conditions to provide empirical evidence for the application of EPO clinically.

## Methods

### Animals

Eighty male Sprague–Dawley rats (8 wks, 180~220 g) were used in this experiment. The animals were purchased from Zoology Institution of Hunan Agricultural University and raised in room temperature with natural illumination and ventilation. Their general health state and activity were monitored closely during the experiment. The experimental protocol for this study was pre-approved by the Hunan Normal University Institutional Review Board and in accord with the Public Health Service policy on the use of animals for research [10].

### Experimental procedure

All the rats went through the adaptation training for one week by running on a horizontal-treadmill (slope gradient 0%, ZH-PT-1 Treadmill, Bio Equipment Co., Ltd. Huaibei, Anhui, China) at a speed of 15–30 m/min for 15–20 min every day. They were grouped randomly into 4 groups of 20 animals: control group (C), exhaustive exercise group (ET), exhaustive exercise + rhEPO group (ET+EPO) and exhaustive exercise+ rhEPO + LY294002(PI-3- kinase inhibitor)group (ET+EPO+LY). Rats of group (ET+EPO) received intraperitoneal (ip) injection of 2000 u/kg rhEPO 30 minutes prior to the exercise every Monday, Wednesday and Friday. With the same schedules and EPO administration, rats of group (ET+EPO+LY) had an extra ip injection of 100 mg/kg LY294002 one hour prior to exercise. The over-training model was based on the modified exercise protocol proposed by Somani [11]. Animals of group ET, ET+EPO and ET+EPO+LY were imposed a treadmill running once a day repeatedly for four weeks at gradient 10% and speed of 30 m/min, simultaneously stimulated by sound, light, machinery until they were exhausted, e.g. unmovable, prostrating on the ground and gasping with no response to evade, which usually took 4 – 5 h. The running distance was recorded automatically with machine.

### Samples collection

Twenty-four h after the exhaustive exercise, rats were anaesthetized by ip injection of 10% chloral hydrate (0.3 ml/100 g) and sacrificed by incising of the heart. The kidney was removed immediately for specimens. Nephridial tissue between cortex and medulla was dissected out and fixed with 10% paraformaldehyde. The tissue was sliced into small pieces (< 1 mm^3^) and placed on the microscope slide immersed with glutaraldehyde fixative. The tissues were fixed with 2% paraformaldehyde - 2.5% glutaraldehyde for two h at 4°C, washed for three times (10 min each) with buffer solution of sodium cacodylate (pH 7.2). After dewetting, the tissues were fixed by 1% H_2_OSO_4_ for 2 h at 4°C and washed again, fixed with 0.1 m buffer solution of sodium cacodylate and preserved at 0°C for electron microscopy examination. Same procedurals were applied for rats in control group except that the samples were collected one day after adaptation training.

### Hematoxylin eosin (HE) staining

The fixed tissue was embedded in paraffin and cut into serial sections of 5 μm. The sections were dewaxed with dimethylbenzene and soaked for 2 min in 100%, 95%, 85% and 70% ethanol respectively for gradient rehydration, then washed for 2 min with distilled water and soaked for 15 min in Haematein dye liquor. After swashed with running water, the sections were soaked in 1% hydrochloric acid alcohol till them turned to pink, and swashed again for 15–30 min to make them back to blue. They were colored with 0.5% eosin stain for 1 min and dehydrated with 70%, 85%, 95%, 100% ethyl alcohol respectively, each for 2 min. Again, they were soaked 5 min in dimethylbenzene for two times to make them transparent. Finally, the sections were sealed with neutral balsam and photographed with OLYMPUS BX52 Microscopic imaging system (The JEOL institute, Japan, 40×10 times).

### Electron microscope specimen preparation

The specimens were gradient dehydrated under 4°C with ethyl alcohol, 50% for 10 min, 70% overnight, 90% 10 min and 100% 15 min twice, and replaced at room temperature with propylene epoxide for 15 min twice, 1:1 propylene epoxide and resin for 1 h, 1:4 propylene epoxide and resin for 1 h and pure resin soakage for 2 h, followed by embedding in pure epoxy resin Epon812 and polymerized in thermostat, 35°C for 16 h, 45°C for 8 h, 55°C for 14 h and 60°C for 48 h. The samples were trimmed and cut into serial sections of 60 nm positioned by azure-cyanine staining optical microscope. The sections were double stained with acetic acid uranium and lead nitrate and studied by a Hitachi H2600 Transmission Electron Microscope.

### Statistical analysis

Data were analyzed by using statistical software SPSS (version 17.0 for Windows, SPSS, Chicago, IL, USA). All data are presented as mean ± SD. one-way ANOVA followed by Bonferroni test was used for comparisons. *P* < 0.05 was considered statistically significant.

## Results

### Changes in general condition

Before exercise, all rats were physical normal and appeared active with bright eyes and lustrous hairs. At the end of each exhaustive exercise as group ET, the rats appeared listless with gloomy eyes. They were short of breath and unstable at feet with chest and belly touching the ground, no response to sound and light stimulation, some serious cases even showed conjunctival hemorrhage. All these symptoms were more evident with each passing training day, running shorter and shorter. In the recovery phases after exercise, diet was less and less, the time to free move longer and longer. Rats in group ET+EPO tolerated better to the exhaustive exercise compared with those in group ET as showing in changes of running distance (Table 1). At the end of each training in the corresponded time, their limbs could support the body and had response to sound and light stimulation; in the recovery phases after exercise, they had normal diet and looked more vivacity. After four weeks exhaustive exercise, the rats weight of control and EP+EPO groups was increased much more than those of rats in ET group (*P* < 0.05).

**Table 1:** Changes in running distance of the rats in four weeks exhaustive exercise (m)

**Group**	**1**^**st **^**week**	**2**^**nd **^**week**	**3**^**rd **^**week**	**4**^**th **^**week**
ET	1640 ± 890*	1890 ± 420*	1380 ± 420*	750 ± 308**
ET+EPO	1590 ± 590*	1860 ± 390*	1509 ± 470*^★^	1270 ± 701**^★★^

Values are expressed as mean ± SD. *P < 0.05, ** *P* < 0.01, difference between training weeks in each group; ^★^*P* < 0.05, ^★★^*P* < 0.01, difference between groups in each corresponded training periods (n = 20, one-way ANOVA followed by Bonferroni test).

### Changes in running distance

The running distances in rats of both ET and ET+EPO groups were significantly shorter week by week after 2nd week training (*P* < 0.05, and *P* < 0.01 between 2^nd^ week and 4^th^ week), while rats of ET+EPO group running much longer than rats of ET group in the training of 3^rd^ week (*P* < 0.05) and 4^th^ week (*P* < 0.01, Table 1).

### Changes in HE renal tissue

**Figure 1:**
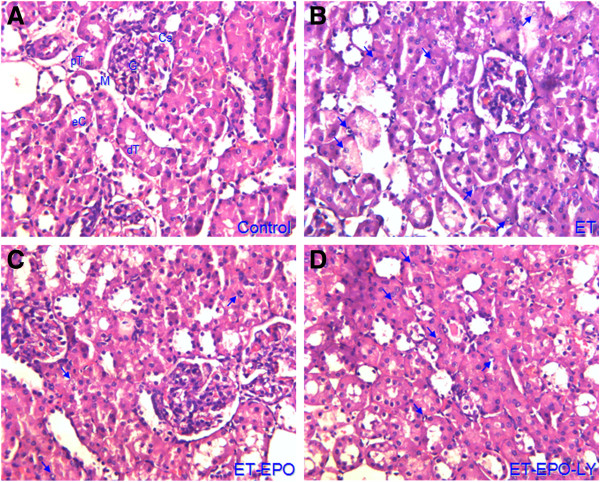
**Representative images of renal tissue stained by HE. A**, rat from control group, (Cs) capsular space, (dT) distal convoluted tubule, (eC) tubule epithelial cell, (G) renal glomerulus, (Bm) basilar membrane; (pT) proximal convoluted tubule; **B**, rat from exhaustive exercise group; **C**, rat from exhaustive exercise plus EPO intervention; **D**, rat from exhaustive exercise plus EPO and LY294002 intervention. Arrows in b to d point to the nucleus of apoptotic cells (×400, the same indications for the following figures).

In rats of control group (Figure 1A), the glomerular extracellular matrix and mesangial cells presented normal distribution, the capillary lumens opened well, the kidney tubules and interstitial region were clear in structure, the epithelial cells in the kidney tubule were square, aligned well with the same size; no fibrous tissue proliferation showed in mesenchyme. In contrast, the rats in ET group showed obvious congested and swollen glomeruli. The renal capsule was narrow. The tubule epithelial cells were disintegrated, denatured and dropped off. The tubule lumens were dilated containing albumen and erythrocyte cast. The tubule walls were stripped and cracked. The interstitial tissue of proximal convoluted tubule presented a remarkable fibrosis. A lot of chromatin clumped, edge-set and dyed apoptotic cells (identified by the appearance of brown particles in the nucleus) appeared in proximal and distal convoluted tubule (Figure 1B). Such pathological changes were slighter in the rats of ET+EPO and ET+EPO+LY groups (Figure 1C, D) as less extravasated blood was shown in glomeruli, the basilar membranes were more intact, the denaturated tubule epithelial cells were less. There were a few lumen expansions but the dropped villus and dropped epithelial cells were much less. Albumen and erythrocyte cast were seen occasionally, while the stripped and cracked tubule walls were rarely seen, and the number of apoptotic cell was much less.

### Changes in ultrastructure of kidney cell

**Figure 2:**
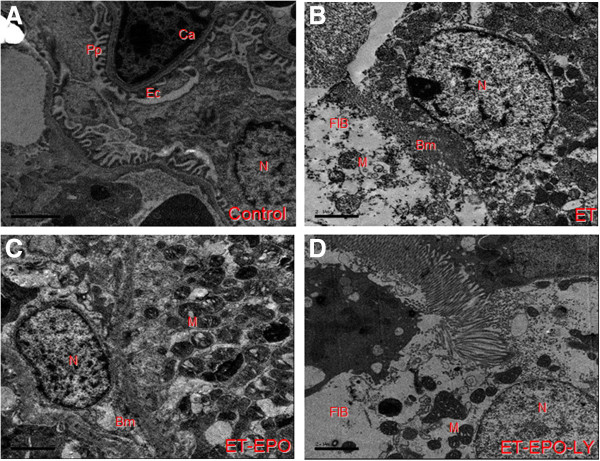
**Representative ultrastructure images of glomerular filtration membrane. A**. rat of control group, **B**. rat of ET group, **C**. rat of ET+EPO group, **D**. rat of ET+EPO+LY group. (Ca) capillaries; (Pp) podocyte processus; (Po) podocyte; (N) neuclei; (Ec) endothelial cell; (Bm) basilar membrane; (FIB) tissue fibrosis; (M) mitochondria; (Ly) lysosome (the same indications for the following figures, Bars are 2 μm).

**Figure 3:**
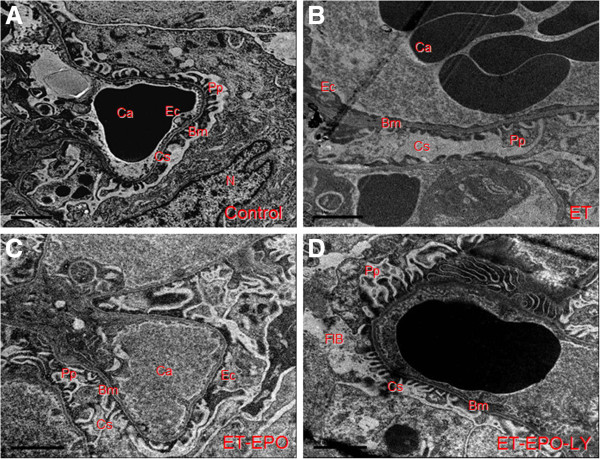
Representative ultrastructure image of kidney cells.

In rat of control group, the endothelial cells (Ec), capsular space (Cs), electron density and the podocyte protuberance (Pp) were clearly visible and evenly distributed (Figure 2A), the thickness of basilar membrane (Bm) was uniform, the karyomorphism was complete, the boundary of the cell membrane and the chromatin were distinguishable (Figures 2A, 3A).

**Figure 4:**
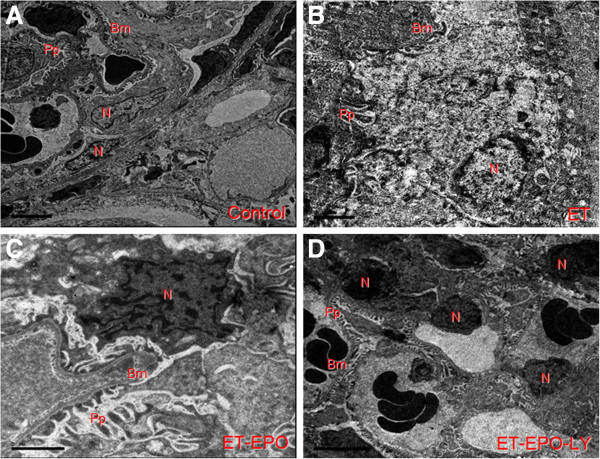
Representative ultrastructure image of nucleus of kidney cells.

However, sample of ET group revealed notable pathological changes in glomerular endothelial cells, manifested as swollen, paired and vaulted cytoplasm, stripped basilar membrane with fused endothelial cells (Figure 2B, 3B). Besides, there were tortuosity and collapse basilar membranes and the three-layer structure were disappear. (Figures 2B, 4B). Changes in glomerular podocyte included that the soma of glomerular periphery loop podocyte was dropped off, foot process fused (Figure 2B), podocyte variation was presented and the number of podocytes were reduced (Figure 3B); the nucleus was incomplete, some of them showed a lobulated apoptosis-like alterations (Figure 4B); the chromatin was clumped and unevenly distributed mainly located in the lower part of nuclear membranes (Figures 2B, 4B). The capsular space (Cs) was irregular (Figure 2B) or much widened (Figure 3B). The mesenchyme of kidney tubule showed massive fibrosis, thus the three-layer structure of filtration membrane was undistinguishable (Figure 2B).

Compared with ET group, samples of ET+EPO group of the rats showed much less ultrastructure abnormalities in renal cells. The endothelial cells (Ec) and renal podocyte protuberance (Pp) were more evenly distributed, the thickness of basilar membrane (Bm) was more identical with clearer boundary (Figure 3C), while the boundary of nucleus was obscure and leaflet change could be seen occasionally (Figure 4C); mitochondria ridge could be seen clearly with part of vacuolization; microvillus was rare observed; no evident mesenchyme fibrosis was shown (Figure 2C).

Samples of ET+EPO+LY group showed apparently damage in kidney cells but lighter than those in ET group of the rats. The podocyte revealed microvilli alteration, mesenchyme fibrosis (Figure 2D) and plentiful leaflet changes of cell nucleus (Figure 4D ).

### Changes in Ultrastructure of Kidney Tubule Epithelial Cell

**Figure 5:**
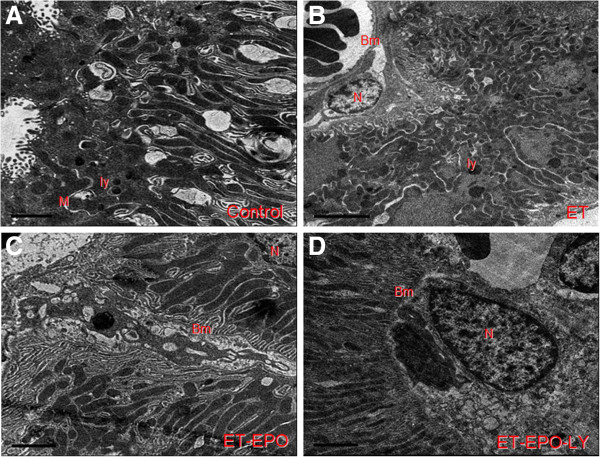
Representative ultrastructure image of epithelial cells on kidney tubule.

The ultrastructure of epithelial cells of distal convoluted tubule in control group was integrated and distinguishable (Figure 5A). The mitochondria (M) were complete with clearly visible ridge and lysosome (Ly). In ET and ET+EPO+LY groups, the epithelial cells appeared remarkable variation (Figure 5B, 5D), mitochondria were shrinking with obscured ridge and boundary; the regions of deep electron density were obviously enlarged in the neuclei (Figure 5D) and apoptotic cells with nucleoplasm condensed in the edges of cell membrane were more evident; basilar membrane showed a noticeable variation and even dissolved, some of the basal infoldings were hard to identify. Compared with ET group, the damage in tubule epithelial cells in rats of ET+EPO group were slighter (Figure 5B), in that the mitochondria boundaries were much clearer with an observable basal infoldings. The basilar membranes were thicker (Figure 5C).

## Discussion

The general conditions was strongly affected by the long last repeated exhaustive exercise as shown in ET group of the rats. The symptoms of the exhaustive exercise were evident and more serious with the continuation of the training days, running shorter, dieting less, body weight decreasing, etc. The running distance was significantly shorter. A considerable damage in kidney cells were presented in histological and ultrastructural examinations. However, treatment with EPO during the training, the general conditions were much better and the pathological changes in kidney cells were remarkable less compared with the ET rats without EPO intervention. The findings suggested a severe chronic renal injury were induced by four-week exhaustive exercise and such a pathological process was counteracted by the EPO intervention. In agreement, similar results had been reported with acute exhaustive exercise [12,13].

Normally, the blood flow in the kidney is about 25% of the cardiac output. When exercise, the metabolic rate of muscle is increased and more blood supply required. In exhaustive situation, blood flow redistribution among the organs occurs due to the emergency response, which is activated by increasing in secretion of adrenaline and rennin angiotensin. Consequently, the renal artery contracted and the renal blood flow decreased, eventually the renal injury aggravated, especially when the exercise lasts and the intensity increase further on. This incomplete ischemia state forms so-called “exercise induced renal ischemia”, which then turns to “ischemia-reperfusion (I/R)” when blood supply regained after cessation of the exercise [14,15]. It was proposed that such a renal injury as we showed in the ET rats is a dynamic process mediated by multiple factors, multiple ways and multiple aspects. The mechanisms were mainly related to the effect of reactive oxygen, intracellular calcium overload, inflammatory corpuscle, inflammatory cascade reaction resulted from combined action of various cell factors and adhesion molecules, cell apoptosis, nitric oxide, endothelin, etc. [14,16].

An intact filtration barrier and proper function of tubular are critical for kidney in maintaining stability of the internal environment. The ultrastructure alternations of renal cells as we found in ET rats were pronounced e.g. disorganized three layers structure of glomerular filtration membrane, mesenchyme fibrosis and cell apoptosis, etc. Since the glomerular endothelial cells are the first layer of filtration barrier, which directly contacts blood flow, affected by the changes in blood component, harmed by hypertransfusion, high filtration and high pressure in the glomeruli [17], this pathological state would weak the cells ability to synthesize hydridic glycoprotein and impair the negative charged barrier [18], in turn aggravate the renal dysfunction.

The morphological abnormalities of glomerular basement membrane (GBM) are another characteristic of renal injury, including unnatural thickness (thinner or thicker), losing of completeness, cracking and immune complex deposition etc., which presented almost all in the present samples of the ET rats. The glomerular podocyte foot process composes glomerular hiatus membrane which is an important filtration barrier for large molecules protein [19]. When width of podocytes foot-process increases, the slit pore number of GBM per unit length decreases, as a result, the mechanical integrity of the barrier would be jeopardized [20]. The present founding that the foot-process fusion or reduction of podocytes in the samples of ET rat further suggesting that glomerular filtration function was impaired after four weeks repeatedly exhaustive excise.

Hypoxia can cause kidney tissue atrophy and fibrosis [21,22]. In agreement, the ET group of the rat in this experiment showed a remarkable fibrosis of renal mesenchyme, which could be due to in short of blood and oxygen supply resulted from blood flow redistribution after long and high intensity exercise. Such a hypoxia might activate fibroblast multiplication and produce fiberized tissues. In addition, the vascular contraction and hypoxia induced vascular wall injuries were another cause of chronic ischemic injury of tubulointerstitial, since the vascular wall injury would results in insufficient oxygen supply and thus the development of tubulointerstitial cells atrophy and fibrosis formation.

Studies showed that EPO could reduce the cerebral ischemia, diminish the cerebral infarction area [5,23], and improve the renal function harmed by the ischemia/reperfusion [24,25]. Recent experiments showed that the protect effect of EPO against renal injury was via promoting the regeneration of tubular epithelial cells and resisting the cell apoptosis [26]. Supportably, we demonstrated here that the ultrastructure abnormalities of renal cell in rat of ET+EPO group were much less than ET rat without EPO intervention. Manifested as the endothelial cells and renal podocytes processes were more uniformly distributed, the basilar membrane boundaries were comparably clearer, the mitochondria ridges were more visible and the mesenchyme fibrosis were barely seen.

Renal tubular injury and fibrosis are two common paths to various causes of renal chronical disease [27]. As to the ET +EPO rats, although apoptotic nucleus could be seen occasionally, there were no notable mesenchyme fibrosis, indicating that the protective effect of EPO is related to the inhibition of cell apoptosis and reduction of interstitial fibrosis formation. However, when LY294002 was applied to the rats as in ET+EPO+ LY group, the ultrastructure abnormalities of renal cell were more pronounced than those in the rats of ET+EPO group, e.g. there were irregular microvilli in the podocytes, a large number of fibrosis and apoptotic changed cells in the tubule mesenchyme. LY294002 is a widely used specific inhibitor of phospholipids phthalocyanine inositol 3-kinase (PI3K) [28,29]. By inhibiting the catalytic activity of PI3K PllO subunit and impeding the production of downstream substrate PIP3, the involved signal pathway will be in an inactivated state [30]. Therefore, we proposed that PI3K/Akt signal pathway takes part in the endogenous protection of kidney damage caused by exhaustive exercise.

When the pathway was blocked by PI3K specific inhibitor LY294002 as we applied in the rats of ET+EPO+LY group, the tolerance of kidney cell to the chronic ischemia and hypoxia was weakened considerably so that the apoptosis cells and interstitial fibrosis produced, which might be the explanations of the present finding that the renal tissue injury was aggravated by the application of LY294002. On the other hand, the protective effect of EPO could be speculated as the activation of PI3K/Akt pathway, and thus increase the kidney resistant to injury and alleviate the secondary damage from chronic ischemia and hypoxia.

## Conclusions

The protective effect of EPO on chronic renal injury induced by repeated exhaustive exercise demonstrated in the present study could be due to the inhibition of cell apoptosis and interstitial fibrosis formation by activating PI3K/Akt pathway, thus plays role in the endogenous protection of the kidney injury. The EPO effect against exhaustive exercise-induced chronic renal injury demonstrated in the present in vivo study in rat points to the potential interest of EPO in such a clinical situation.

## Competing interests

The authors declare that they have no competing interests.

## Authors’ contributions

XXL, CHJ and SLQ conceived the study, established the design and carried out the experimental work. CHJ and ZQL participated in the data analysis and provided critical comments on the study design and manuscript. XXL and CHJ drafted this manuscript. All authors read and approved the final manuscript.

## Authors’ information

Xixiu Lin and Chonghe Jiang are co-authors.

## Pre-publication history

The pre-publication history for this paper can be accessed here:

http://www.biomedcentral.com/1471-2369/14/130/prepub
